# Bacterial Natural Disaccharide (Trehalose Tetraester): Molecular Modeling and in Vitro Study of Anticancer Activity on Breast Cancer Cells

**DOI:** 10.3390/polym12020499

**Published:** 2020-02-24

**Authors:** Biliana Nikolova, Georgi Antov, Severina Semkova, Iana Tsoneva, Nelly Christova, Lilyana Nacheva, Proletina Kardaleva, Silvia Angelova, Ivanka Stoineva, Juliana Ivanova, Ivanina Vasileva, Lyudmila Kabaivanova

**Affiliations:** 1 Institute of Biophysics and Biomedical Engineering, Bulgarian Academy of Sciences, “Acad. G. Bonchev” Str., Bl. 21, 1113 Sofia, Bulgaria; antov8107@abv.bg (G.A.); severina.yordanova@gmail.com (S.S.); itsoneva@bio21.bas.bg (I.T.); 2 Institute of Plant Physiology and Genetics Bulgarian Academy of Sciences, “Acad. G. Bonchev” Str., Bl. 21, 1113 Sofia, Bulgaria; juivanova@yahoo.com (J.I.); ivanina_vasileva1@abv.bg (I.V.); 3 The Stephan Angeloff Institute of Microbiology, Bulgarian Academy of Sciences, “Acad. G. Bonchev” Str., Bl. 26, 1113 Sofia, Bulgaria; nchrist@abv.bg (N.C.); lin1@abv.bg (L.N.); 4 Institute of Organic Chemistry with Centre of Phytochemistry, Bulgarian Academy of Sciences, “Acad. G. Bonchev” Str., Bl. 9, 1113 Sofia, Bulgaria; bastet@gbg.bg (P.K.); istoineva@yahoo.com (I.S.); 5 Institute of Optical Materials and Technologies “Acad. Jordan Malinowski”, Bulgarian Academy of Sciences, Sofia, “Acad. G. Bonchev” Str., Bl. 109, 1113 Sofia, Bulgaria; sea@iomt.bas.bg

**Keywords:** bacterial natural disaccharide, trehalose tetraester, *Rhodococcus wratislaviensis*, breast cancer, anticancer activity, molecular modeling

## Abstract

Isolation and characterization of new biologically active substances affecting cancer cells is an important issue of fundamental research in biomedicine. Trehalose lipid was isolated from *Rhodococcus wratislaviensis* strain and purified by liquid chromatography. The effect of trehalose lipid on cell viability and migration, together with colony forming assays, were performed on two breast cancer (MCF7—low metastatic; MDA-MB231—high metastatic) and one “normal” (MCF10A) cell lines. Molecular modeling that details the structure of the neutral and anionic form (more stable at physiological pH) of the tetraester was carried out. The tentative sizes of the hydrophilic (7.5 Å) and hydrophobic (12.5 Å) portions of the molecule were also determined. Thus, the used trehalose lipid is supposed to interact as a single molecule. The changes in morphology, adhesion, viability, migration, and the possibility of forming colonies in cancer cell lines induced after treatment with trehalose lipid were found to be dose and time dependent. Based on the theoretical calculations, a possible mechanism of action and membrane asymmetry between outer and inner monolayers of the bilayer resulting in endosome formation were suggested. Initial data suggest a mechanism of antitumor activity of the purified trehalose lipid and its potential for biomedical application.

## 1. Introduction

The isolation, characterization, and study of the properties of new bioactive compounds are a crucial part of the fundamental research in the field of biology and medicine and provide unlimited opportunities for new drug discoveries [1,2].

Biosurfactants are naturally occurring surface-active biomolecules produced by a variety of microorganisms [3,4,5,6]. 

Usually, biosurfactants have an amphiphilic character, with molecules possessing both hydrophilic and lipophilic/hydrophobic components/moieties, which is a predisposition for their unique biological properties. Microorganisms produce many different, in some cases unique, amphiphilic metabolites that can be classified into several groups based on their chemical structure: (i) glycolipids, (ii) phospholipids, (iii) lipopeptides and lipoproteins, (iv) fatty acids, (v) neutral acids, and (vi) polymeric and particulate surfactants [5,6]. Glycolipids are the most commonly used biosurfactants. Trehalolipids, or trehalose lipids (TL), synthesized by most species belonging to the mycolates group of microorganisms [3,4,5,6], are glycolipids containing a trehalose carbohydrate moiety. The hydrophilic trehalose moiety is combined with long-chain aliphatic or hydroxyl acids and linked with an ester moiety.

Due to their structural and functional diversity, biosurfactants appear to be an attractive group of molecules with great potential to be used in numerous industrial, biotechnological, and medical applications [7,8,9,10].

Recently, trehalose lipids were proved to be a good alternative to conventional medicines. Due to their surface activity, they could be considered as possible anticancer therapeutics [11,12]. According to published data, in vitro cell death in various human carcinomas has been triggered after treatment with TL biosurfactant [11,12,13].

**Scheme 1:**
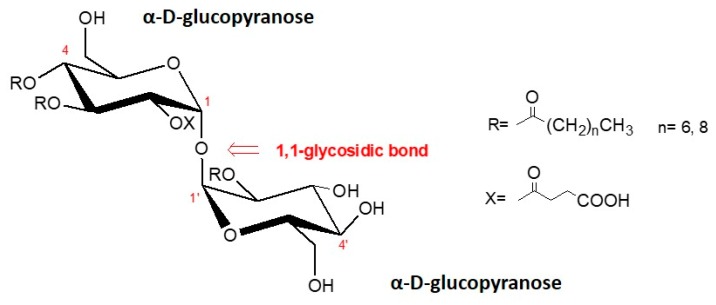
Chemical structure of the *Rhodococcus sp*. trehalose lipid.

However, the effect of trehalose lipid (Scheme 1) on the changes in morphology, viability, proliferation, and migration ability of breast cancer has not been studied in depth.

The objective of this work is to isolate glycolipid biosurfactants produced by *Rhodococcus wratislaviensis* strain BN38, to model their conformation at physiological pH and to determine the tentative sizes of the hydrophilic and hydrophobic portions. In addition, the antitumor activity of the anion form of trehalose lipid tetraester against human breast cancer cell lines was evaluated, regarding cell viability, migration, and clonogenic capacity.

## 2. Materials and Methods

### 2.1. Bacterial Strain and Cultivation

The biosurfactant-producing strain *Rhodococcus wratislaviensis* BN38 used in this study was isolated and taxonomically identified as described previously [14]. The strain was cultivated in mineral salt medium, containing in (g L^−1^): K_2_HPO_4_ •3H_2_O (4.8); KH_2_PO_4_ (1.5); (NH_4_)_2_SO_4_ (1.0); Na_3_(C_6_H_5_O_7_) 2H_2_O (0.5); MgSO_4_ • 7H_2_O (0.2); yeast extract (0.1), supplemented with trace element solution with the following composition in (mg L^−1^): CaCl_2_ • 2H_2_O (2.0); MnCl_2_ 4H_2_O (0.4); NiCl_2_ •6H_2_O (0.4); ZnSO_4_ •7H_2_O (0.4); FeCl_3_ •6H_2_O (0.2); Na_2_MoO_4_ • 2H_2_O (0.2), and 2% n-hexadecane. The medium was shaken at 120 rpm for 7 days at 28 °C.

### 2.2. Detection of Biosurfactant Production

Surface tension (ST) measurement and emulsifying activity were used to screen biosurfactant production. Samples of the culture media of the selected strain were centrifuged at 8000 *g* for 20 min. Surface tension of the supernatant fluid of the culture was measured by the ring method using a DuNouy ring tensiometer (Kruss T 10, Hamburg, Germany). The emulsifying activity of the culture supernatant was estimated by adding 0.5 mL of sample fluid and 0.5 mL of kerosene to 4.0 mL of distilled water. The tube was vortexed for 10 s, held still for 1 min, and then examined for turbidity of a stable emulsion.

### 2.3. Purification

The surface-active compounds were isolated by liquid−liquid extraction with methyl-tert-butyl ether. The isolated products were analyzed by thin layer chromatography on silica gel G60, using chloroform/methanol/water (65:15:2), as mobile phase, and visualized with a sugar-specific reagent anthrone (10H-anthracene-9-one) in sulfuric acid and heated to 150 °C for 2–3 min. The crude glycolipid mixture was dissolved in chloroform/methanol/water (65:15:2, *v*/*v*/*v*) and afterward separated by using medium-pressure liquid chromatography.

The obtained main fractions with surface activity were identified with ^1^H NMR, ^13^C NMR, and ESI-MS analysis as succinoyl 2,3,4,2′-trehalose tetraester with molecular mass of 876 and 848, respectively.

### 2.4. Computational Details

The molecular modeling of the structure and possible interactions between two molecules of TL was carried out using density functional theory (DFT) calculations. DFT is a powerful method capable of accurate results at a relatively low cost. Geometry optimization was carried out at the B3LYP/6-31G (d, p) level of theory. The crystal structure of α, α-trehalose (Cambridge Crystallographic Data Centre (CCDC) identifier DEKYEX, deposition number 1138536) [15] was used in the modeling of the trehalose tetraester. Ester R fragments with 6 and 8 CH_2_-groups (1st glucose) and 8 CH_2_-groups (2nd glucose) were attached to the parent α, α-trehalose. The neutral and the anionic form with one negative charge on the X carboxylic group, corresponding to the pH 7 structure of this trehalose tetraester were considered. After geometry optimization, frequency calculations were performed at the same level of theory. No imaginary frequency was found for the lowest energy configurations of any of the optimized structures.

The differences ΔEel, ΔEth, PΔV (work term), and ΔS between the product (TL dimer) and reactant (TL) were used to evaluate the gas-phase free energy of the complex formation, ΔG, at T = 298.15 K according to the equation:

ΔG = ΔEel + ΔEth + PΔV − TΔS.(1)

The complex formation is thermodynamically favorable if the calculated value of ΔG is negative, and the process is unfavorable if the value of ΔG is positive.

Solvation effects were accounted for by employing the solvation model based on density (SMD) [16] scheme and performing single point calculations in water (ε = 78).

Gaussian 09 suites of programs were used for all calculations [17].

Molecular graphics images were made using PyMOL [18].

### 2.5. Cell Lines and Culture Conditions

All cell lines (MCF-10A, MCF-7, and MDA-MB-231) were purchased from the American Type Culture Collection—ATCC (Manassas, VA, USA). The human breast adenocarcinoma cell lines (MCF-7 low-metastatic and MDA-MB-231 high-metastatic) were cultivated in Dulbecco’s modified Eagle’s medium (DMEM) supplemented with 10% fetal bovine serum (FBS), 1% sodium pyruvate, 1% MEM non-essential amino acids (NEAA) without antibiotics. As control in all experiments, the non-tumorigenic breast epithelial cell line (MCF-10A) were cultivated in DMEM medium (Sigma-Aldrich, St. Louis, MO, USA) supplemented with 10% FBS, 1% sodium pyruvate, 1% non-essential amino acids (NEAA), 20 ng/mL human epithelial growth factor (hEGF), 10 μg/mL insulin, 0.5 μg/mL hydrocortisone without antibiotics. All cell lines were maintained in a humidified atmosphere with 5% CO_2_ at 37 °C.

### 2.6. Trehalose Lipid Tetraester (TL)

The isolated TL from *R. wratislaviensis* was dissolved in DMSO (Eurobio Scientific, Les Ulis, France) to obtain a master stock solution at a concentration of 5.7  mM (5 mg/mL) and stored at 4 °C. The working solution (570 µM) was freshly prepared prior to each experiment in the appropriate culture medium. Final DMSO concentrations in each sample were calculated previously (lower than 1%) to provide absence of DMSO—relative toxicity.

### 2.7. Cell Viability (MTT) Assay

The cytotoxic activity of TL on the cultured cells was measured by an MTT (3-(4,5-dimethylthiazol-2-yl)-2,5-diphenyl tetrazolium bromide) colorimetric test [19]. The cells were grown in 96-well plates at a density of 1 × 10^5^ cells/mL. After overnight incubation, the cells were treated with different concentrations (from 10 to 100 µM) of the test compound and incubated for 24 and 48 h. Later, the MTT solution (20 µL of 5 mg/mL, Sigma-Aldrich, St. Louis, MO, USA) was added to each well, and the plates were re-incubated for an additional 4 h. Finally, the medium was removed and 100 µL of lysis buffer (10% SDS, 0.01 M HCl) was added to solubilize the formed formazan crystals. The amount of formazan crystal was determined by measuring the absorbance at 570 nm using a Thermo Multiskan Spectrum spectrophotometer (Thermo Fisher Scientific, Waltham, MA, USA) and the half-maximal inhibitory concentration (IC_50_) of the pure compound was calculated by GraphPad software. All experiments were performed in triplicate, at least three times independently.

### 2.8. Wound Healing (WH) Assay

MCF10A, MCF7, and MDA-MB231 cells (7 × 10^5^ cells/well) were seeded in a CytoSelect™ 24-well Wound Healing Assay plate containing 12 wound field inserts (Cell Biolabs Inc., San Diego, CA, USA) and allowed to adhere overnight. After the adherent period, the wound field inserts were removed, and the detached cells were washed three times with 500 μL of sterile PBS (1×). Fresh medium (500 μL), containing 75 μM of TL was added afterward, and wound healing was maintained for 24 h. Cell migration was monitored and documented under phase-contrast microscope Carl Zeiss Jena (Zeiss, Berlin, Germany) with 10× magnification at 0 and 24 h post-induction of wound area [20]. Wound closure was measured and quantified using Image J software version 1.8.0_112 [21]. The experiment was repeated at least two times.

### 2.9. Colony Forming Cell (CFC) Assay

The in vitro cell survival assay was performed with minor modifications according to Franken et al. [22]. In brief, MCF10A, MCF7, and MDA-MB231 cells were seeded in 6-well plates at a density of 500 cells/mL. The cells were allowed to adhere overnight and treated the next day with the selected concentration of TL. After 9 days of incubation period, the medium was removed, and cell colonies were stained with a 2% solution of methylene blue in 50% ethanol [23], and all colonies consisting of more than 50 cells were enumerated.

### 2.10. Cell Morphology Imaging

To assess changes in cell morphology, all cell lines (treated and untreated) were examined under phase-contrast light microscope (Zeiss, Berlin, Germany). Furthermore, all cell cultures were monitored for the presence of cytopathic effect (CPE).

### 2.11. Statistical Analysis

All statistical analyses were performed using Microsoft Excel Software. Data are reported as means ±SD. To evaluate the statistical significance of experimental data, comparisons between treated and control probes and cancer and normal cell lines were performed by Student’s *t*-test. Each P-value lower than 0.05 was considered statistically significant.

## 3. Results and Discussion

Biosurfactants represent a broad group of natural disaccharides with bacterial origin with a promising potential for biomedical applications. In this study, we tested the anticancer effect of TL applied at different concentrations (10–100 µM) for 24 and 48 h against two types of breast cancer cell lines high-metastatic MDA-MB231 and low-metastatic MCF7, as well as against the control non-cancer epithelial cell line MCF10A.

**Figure 1:**
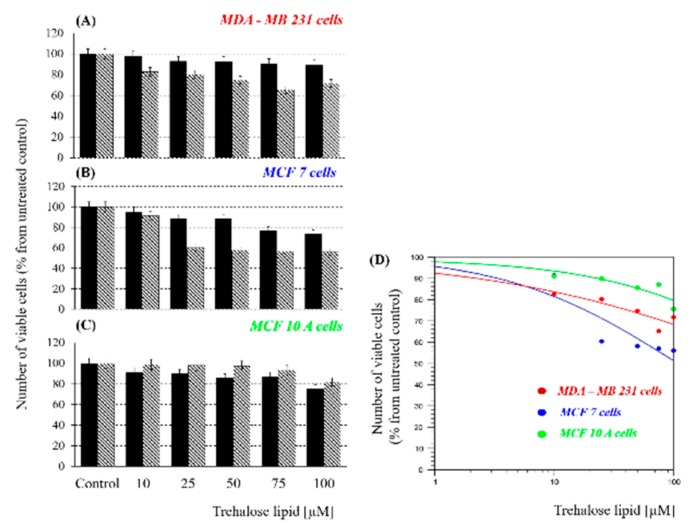
Number of viable cells after treatment with trehalose lipids (TL) at different concentrations (10, 25, 50, 75, 100 µM), presented as a percentage of control (untreated) cells, analyzed by MTT assay. Presented in (**A**–**C**) results are respectively for: MDA–MB23, MCF7 cancer cells, and normal MCF10A cell line; black solid columns present data after 24 h incubation and the striped columns after 48 h. All data are means ±SD from three independent experiments. Statistical analysis: (i) All results are statistically significant versus controls at corresponding incubation time (*p*-value <0.05); (ii) All presented data from 24 h are statistically significant versus data from 48 h (*p*-value <0.01); (iii) Cancer lines versus Normal cells: all variations between data in (**A**) are significant versus data in (**C**): *p*-value <0.001 for both 24/24 and 48/48 h type comparison; all variations between data in (**B**) are significant versus data in (**C**): *p*-value <0.05 for 24/24 h and *p*-value <0.01 for 48/48 h comparison. (iv) Data from cancer cell lines’ comparison were calculated also as a statistically significant result (*p*-value <0.01 for both 24/24 and 48/48 h variations). (**D**) GraphPad data calculation of the half-maximal inhibitory concentration (IC_50_)—number of viable cells 48 h after treatment with TL in different concentrations, as a percentage of control (untreated) cells. Values are means ±SD from at least three independent experiments at triplet repetitions.

Figure 1A–C shows the cell viability obtained for all cell lines 24/48 h after treatment. Negative controls (without any treatment) were included in the figures as 100%. As shown in Figure 1B, the highest treatment effect was found for the low-metastatic cell line MCF7, followed by high-metastatic MDA-MB231 (Figure 1A), and almost no effect on the normal line MCF10A (Figure 1C). This result differs from the usual one in which the highly metastatic MDA-MB231 cell line is more strongly affected. In Figure 1D are presented the GraphPad calculations of the IC_50_ doses at 48 h: MDA-MB231—770 µM; MCF7—108 µM, and healthy MCF10A cells—1026 µM, respectively.

The results show decrease in the percentage of viable cells with increasing TL concentrations and exposure times, thus suggesting the cytostatic/cytotoxic effect of trehalose lipid on the treated cancer cell lines. It is interesting to note that the viability of the normal MCF10A cell line, which is less affected by TL at concentrations of 10–50 µM after 48 h treatment, has an increased viability of up to 100% (including standard deviation) compared to the treatment for 24 h which is probably related to an increase in the survival rate. A similar dependence was also observed after treatment with TL at concentrations of 75 and 100 µM for 48 h, even though the decrease of the survival rate was relatively low (10%–20%, respectively). Moreover, the absence of strong influence on the survival rate of normal cells and on the other hand decreasing of cancer cell viability, but not more than 45%, is a very promising result for future studies and applications of TL in chemotherapy (for example in combination with conventional anticancer drugs).

The next step of our study was to analyze the migration capacity of the cells after TL treatment. A wound healing assay is a laboratory technique used to study cell migration and cell–cell interaction. The cell migration is a hallmark of wound repair, cancer invasion and metastasis, angiogenesis, etc. Cell invasion and migration are involved in the dissemination of cancer cells, which is the main cause of resistance to treatment and can lead to locoregional or metastatic recurrence after cancer treatment [24]. In vitro analysis of the cell migration is useful to follow alterations in cell migratory potential as a response to experimental treatments. Since IC_50_ was not reached for all cell lines, we chose 75 µM as an appropriate subcytotoxic concentration for further analysis (cell migration and colony formation). At this concentration, all cell lines showed the maximum decrease in cell survival (see data in Figure 1).

**Figure 2:**
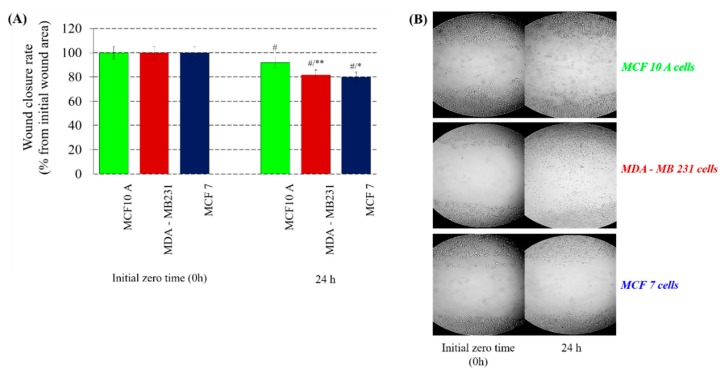
(**A**) Wound closure rate of breast normal/cancer cells 24 h after treatment with subcytotoxic concentration TL (75 µM), analyzed by CytoSelect™—wound healing (WH) assay. Calculated wound area of MCF10A normal cells (green columns), high-metastatic MDA-MB231 cell line (red columns) and low-metastatic MCF7cell line (blue columns) are presented as a percent of initial wound area at the initial zero time. All data are the means ±SD from two independent experiments and three independent calculations via ImageJ software; *p < 0.05 versus MCF10A normal cells, ** *p* < 0.01 versus MCF10A normal cells; # *p* < 0.05—all variations versus corresponding controls are statistically significant. (**B**) Cell migration capacity of normal MCF10A cells (top panel) and cancer cells (high-metastatic MDA-MB231 cells—middle panel; low-metastatic MCF7 cells—bottom panel) after treatment with subcytotoxic concentration (WH assay). Images were obtained after 24 h incubation by phase-contrast microscopy, magnification 10×.

After 24 h of TL treatment, a widening of the gap was detected in all cell cultures (Figure 2B).

However, the strongest effect of TL was observed on the low-metastatic MCF7 cell line (Figure 2B—bottom panel), which can be explained by the effect of TL on the cell membrane and is in agreement with the data from the MTT assay (Figure 1). Moreover, in this case there are qualitative morphological changes—visibly rounded cells compared to the controls (0 h) as shown in Figure 2B—bottom panel.

It could be suggested that this is due to the biosurfactant nature of TL, which together with the survival data obtained, is a prerequisite to believe that it has a cytostatic effect rather than cytotoxic—cells stop dividing but are still vital at subcytotoxic concentrations.

On Figure 2A the same results are calculated in percentage by using Image J software version 1.8.0_112. As can be seen from Figure 2A, the gap filling rates, expressed in percentage, are very close between the two cancer lines but statistically significant. It should be noted that the treatment concentration of TL (75 µM) for the low-metastatic cell line is very close to the value of IC_50_ (108 µM), while for the high-metastatic cell line it is almost ten times lower than IC_50_ (770 µM). In this case, cell lines with different metastatic potential reacted in a similar manner after treatment with the same concentration (75 µM). That suggests a different mode of action of TL on different types of cells. At an equal number of biosurfactant molecules per single cell, surface modification of the cell membrane is similarly expressed by cytoskeleton reorganization followed by suppressing the migration. According to Sarkar et al. [25], trehalose does not readily cross the cell membrane but can be efficiently loaded into the mammalian cells via endocytosis. Obviously, MDA-MD231 needs more molecules of TL (higher threshold) into cells to be killed.

The next step in the study involved a colony formation test. This method provides information on the effect of long-term treatment on the number of colonies formed after 9 days of treatment with subcytotoxic TL concentration (75 µM). Measuring the ability of an individual cell to form colonies (a population of at least 50 cells) gives a real idea of the number of cells with preserved clonogenicity in the cell population. This method has become the gold standard for assessing cellular sensitivity and for determining the percentage of vital to apoptotic cellular fraction under experimental conditions, both in cell lines and in primary cell cultures.

**Figure 3:**
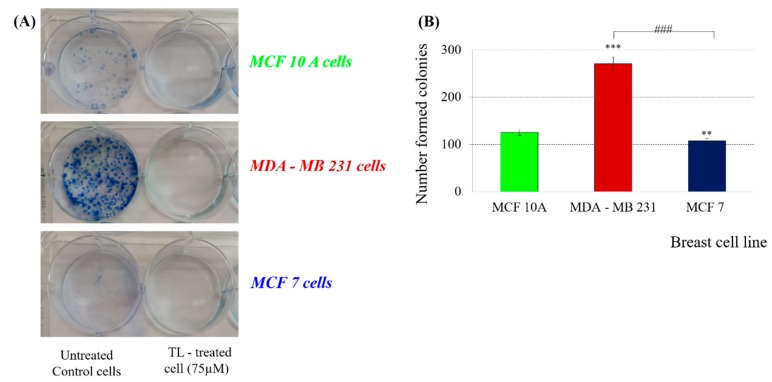
(**A**) Clonogenicity of normal MCF10A cells (top panel) and cancer cells (MDA-MB231 cells—middle panel; MCF7 cells—bottom panel) after long-term treatment with subcytotoxic concentration TL concentration (75 µM), analyzed by colony forming cell (CFC) assay. Untreated cells were used as a control. (**B**) Total number formed colonies of MCF10A, MDA MB231, and MCF7 breast cell lines after long-term treatment with subcytotoxic TL concentration (75 µM). All data are the means ±SD from two independent experiments and three independent enumerations; **p < 0.01 versus MCF10A normal cells, *** *p* < 0.001 versus MCF10A normal cells; ### *p* < 0.001—high-metastatic versus low-metastatic cancer cell line.

A strong anticlonogenic effect was found on all three cell lines after treatment with subcytotoxic concentration (75 µM)—no colonies were found 9 days after treatment compared to untreated control cells (Figure 3).

In our view, this effect is partly due to the alterations in cell morphology, such as changes in the tubulin cytoskeleton, which prevents the formation of the mitotic spindle and subsequent cell division. This explanation is in consistent with our assumption of the existence of cytostatic rather than cytotoxic effect of TL on the tested cell lines.

According to Nasr Eldeen et al. [26], trehalose induces autophagy processes, the first step of which was assumed to be endosome formation. On the other hand, Mackeh et al. [27] reported a relationship between autophagy and microtubules. The authors also point out that more and more anticancer drugs cause autophagy as a cellular opening after treatment. In view of the obtained results (change of cell morphology—rounding of the treated cells) and the references found, we can speculate that the foundations of the molecular mechanism of action of TL are based precisely on the effect of autophagy-mediated cytoskeletal cancer cells by triggering spliced signaling pathways. It is the direction in which future research will continue.

Some theoretical (quantum-chemical) computations were made in order to obtain information for the structure of the studied TL biosurfactant and to shed some light on the possible mechanism of its action in vivo.

**Figure 4:**
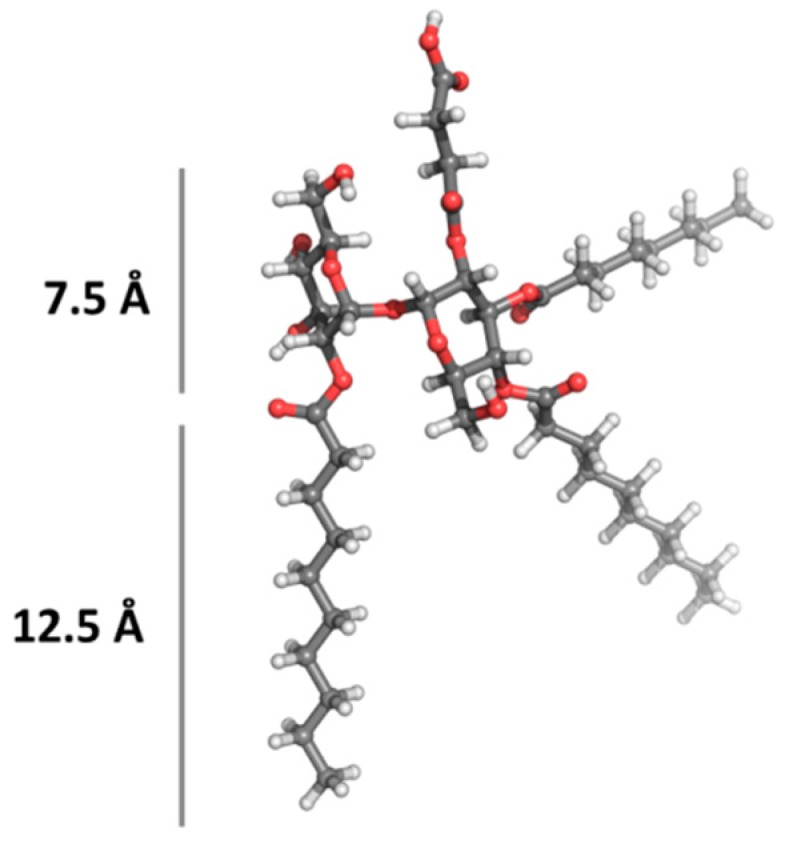
Optimized structure of TL. Molecular modeling was performed as described in the text.

A molecular modeling, which details the size of the hydrophilic and hydrophobic portions of the glycolipid molecule (TL), was carried out. The optimized structure of the most stable rotamer of TL is visualized in Figure 4. The tentative sizes of the hydrophilic (about 7.5 Å) and hydrophobic (about 12.5 Å) portions of the molecule are also shown.

Amphiphiles (both synthetic and natural) are characterized with a strong tendency to self-assemble into a diversity of supramolecular structures/aggregates (micelles, vesicles, nanofibers, nanotubes, etc.) [28]. The possibility of applying biosurfactants in practice as functional materials depends on their self-organizing and self-assembling properties [29].

**Figure 5:**
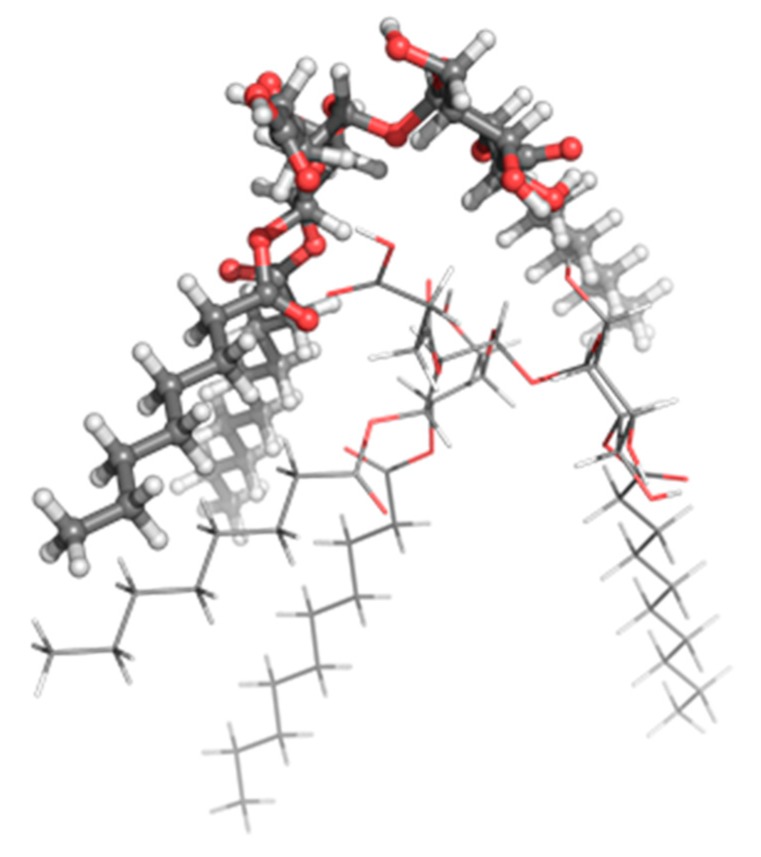
Two (neutral) molecules of TL are shown to depict possible molecular interactions between them. One molecule is presented with sticks for the sake of simplicity.

Molecular modeling of the TL structure and possible aggregation of two TL molecules into a larger unit was carried out using density functional theory (DFT) calculations. The optimized structure of a possible dimer is visualized in Figure 5. TL in (i) its neutral and (ii) non-protonated (anionic) state with one negative charge on the carboxylic group (thus corresponding to the pH 7.4 structures, the pH of extracellular fluid) was taken into account.

A self-assembly process is a spontaneous (exergonic) process that leads toward equilibrium. An exergonic reaction is a reaction where the change in the free energy, ΔG, is negative. For the TL dimer formation reaction from neutral molecules a negative ΔG value (−0.12 kcal mol^-1^) has been calculated at the B3LYP/6-31G (*d*, *p*) level of theory, whereas for the TL dimer formation from TL anions a positive value has been obtained (60.51 kcal mol^-1^). In water environment, the respective ΔG values for the TL dimer formation from neutral and anionic forms are 6.85 and 11.86 kcal mol^-1^, respectively. It can be concluded that at physiological pH 7.4 the self-assembly process for TL in anionic state (with negative charges on the carboxylic groups) is unfavorable at ε = 1 (lipid, approximated by the gas phase) and ε = 78 (water). Thus, the TL in the extracellular fluid is expected to be mostly monomeric. This suggests that the used biosurfactant interacts as a single molecule.

It is interesting that at the same concentration of the used biosurfactant (75 µM that is below the critical micelle concentration—300 µM) [30] the viability, migration, and colony forming responses are different (Figure 1, Figure 2 and Figure 3). This could be due to the “molecular basis” of the action of the trehalose lipid biosurfactant, connected with the physicochemical reorganization of both monolayers of the cell membranes. It is known that the trehalose lipid biosurfactant isolated from *Rhodococcus sp.* could be considered as a weak detergent that prefers membrane incorporation over micellization. According to our theoretical model (Figure 4) the length of used TL is in the range of 20 Å. It is widely known that ions such as K+, with a ion radius between 1.4 and 1.6 Å, freely pass through small pores. That is why it is difficult to suggest that a monomeric biosurfactant such as TL could be transported into the somatic cells through the small pores.

TL, used at a concentration below the critical micelle concentration is a negatively charged (anionic) molecule, which expresses lipid–membrane interaction, due to the pore/defect formation and the leakage of small molecules—small fluorescent rays or K+ ions, through the liposomes or human erythrocytes membrane [30,31]. According Zaragosa et al. [31] the above biosurfactant induces changes in the morphology (positive and negative curvatures) expressed in spherocyte or echinocyte formation. Unfortunately, the suggested “molecular basis” for the mechanism of interaction of TL with erythrocytes could fit partly (surface morphology deformation) to explain the interaction with the use of somatic cells.

Contrary to the erythrocytes, the epithelial breast cells as MCF10A, MDA MB231, and MCF7 have microvilli and the presence of nucleus. On the other hand, the surface electric charges, surface roughness, morphological, internal microenvironment and cytoskeleton network structures, and phospholipid composition of healthy and cancer cells are different (depending on metastatic potential) [32,33,34]. After treatment with TL for both breast cancer cell lines, a strong cytopathic effect accompanied with cell shrinkage and membrane blebbing were observed (Figure S1).

The changes in morphology and migration allow speculation on the following mechanism of interaction: the used monomeric biosurfactant interacts and penetrates into the outer leaflet of the membrane, segregates into different sizes (depending on the surface electrical charges and interactions) and could provoke the appearance of asymmetry between the outer and inner monolayer of the bilayer in the membrane. Such asymmetry in both monolayers could result in membrane invagination and, eventually, endosome formation with different sizes into the cells, depending on their type of malignancy.

## Supplementary Materials

The following are available online at https://www.mdpi.com/2073-4360/12/2/499/s1, Figure S1: Figure S1. Changes in cell morphology of normal MCF10A cells /top panel/ and cancer cells /MDA-MB231 cells – middle panel; MCF7 cells – bottom panel/ 48 h after treatment with subcytotoxic concentration TL concentration (75 μmol), analyzed by phase-contrast light microscopy. Untreated cells were used as a control.

## References

[1] Giddings L.A., Newman D.J. (2013). Microbial natural products: Molecular blueprints for antitumor drugs. J. Ind. Microbiol. Biotechnol..

[2] Chen J., Li W., Yao H., Xu J. (2015). Insights into drug discovery from natural products through structural modification. Fitoterapia.

[3] Delegan Y., Sargsyan A., Hovhannisyan N., Babayan B., Petrikov K., Vainstein M. (2020). Analysis of genome sequence and trehalose lipid production peculiarities of the thermotolerant *Gordonia strain*. J. Basic Microbiol..

[4] Marchant R., Banat I.M., Thavasi R. (2019). The future of microbial biosurfactants and their applications. Microbial Biosurfactants and Their Environmental and Industrial Applications.

[5] Wang Y., Nie M., Diwu Z., Lei Y., Li H., Bai X. (2019). Characterization of trehalose lipids produced by a unique environmental isolate bacterium Rhodococcus qingshengii strain FF. J. Appl. Microbiol..

[6] Kuyukina M.S., Ivshina I.B., Alvarez H. (2019). Production of trehalolipid biosurfactants by Rhodococcus. Biology of Rhodococcus.

[7] Gudina E., Rangarajan V., Sen R., Rodrigues L. (2013). Potential therapeutic applications of biosurfactants. Trend Pharmacol. Sci..

[8] Rodrigues L., Banat I.M., Texeira J., Oliviera R. (2006). Biosurfactants: Potential applications in medicine. J. Antimicrob. Chemother..

[9] Wu Y.-S., Ngai S-Ch Goh B.-H., Chan K.-G., Lee L.-H., Chuah L.-H. (2017). Anticancer activities of surfactin and potential application of nanotechnology assisted surfactin delivery. Front. Pharmacol..

[10] Cherif S. (2019). Biosurfactants in biomedical fields. J. Bacterol. Mycol..

[11] Christova N., Lang S., Wray V., Kaloyanov K., Konstantinov S., Stoineva I. (2015). Production, structural elucidation and in vitro antitumor activity of trehalose lipid biosurfactant from Nocardia farcinica strain. J. Microbiol. Biotechnol..

[12] Duarte C., Gudina E., Lima C., Rodriges L. (2014). Effect of biosurfactants on the viability and proliferation of human breast cancer cells. AMB Express.

[13] Matsumoto Y., Cao E., Ueoka R. (2013). Growth inhibition by novel liposomes including trehalose surfactant against hepatocarcinoma cells along with apoptosis. Anticancer Res..

[14] Tuleva B., Christova N., Cohen R., Stoev G., Stoineva I. (2008). Production and structural elucidation of trehalose tetraesters (biosurfactants) from a novel alkanothrophic Rhodococcus wratislaviensis strain. J. Appl. Microbiol..

[15] Jeffrey G., Nanni R. (1985). The crystal structure of anhydrous α, α-trehalose at −150°. Carbohydr. Res.

[16] Marenich A.V., Cramer C.J., Truhlar D.G. (2009). Universal Solvation Model Based on Solute Electron Density and on a Continuum Model of the Solvent Defined by the Bulk Dielectric Constant and Atomic Surface Tensions. J. Phys. Chem. B.

[17] Frisch M., Trucks G., Schlegel H., Scuseria G., Robb M., Cheeseman J.R., Scalmani G., Barone V., Mennucci B., Petersson G.A. (2013). Gaussian ~09 Revision D.01.

[18] DeLano W.L. (2015). The PyMOL Molecular.

[19] Mosmann T. (1983). Rapid colorimetric assay for cellular growth and survival: Application to proliferation and cytotoxicity assays. J. Immunol. Methods.

[20] Rodriguez L.G., Wu X., Guan J.L., Guan J.L. (2005). Wound-healing assay. Cell Migration.

[21] Rasband W.S. (1997–2018). ImageJ.

[22] Franken N.A., Rodermond H.M., Stap J., Haveman J., van Bree C. (2006). Clonogenic assay of cells in vitro. Nat. Protoc..

[23] Guda K., Natale L., Markowitz S.D. (2007). An improved method for staining cell colonies in clonogenic assays was used. Cytotechnology.

[24] Guy J.B., Espenel S., Vallard A., Battiston-Montagne P., Wozny A.S., Ardail D., Alphonse G., Rancoule C., Rodriguez-Lafrasse C., Magne N. (2017). Evaluation of the cell invasion and migration process: A Comparison of the Video Microscope-based Scratch Wound Assay and the Boyden Chamber Assay. J. Vis. Exp..

[25] Sarkar S., Davies J., Huang Z., Tunnacliffe A., Rubinztein D. (2007). Trehalose, a novel m TOR-independent autophagy enhancer, accelerates the clearance of mutant hunting and alpha-syneclein. J. Biol. Chem..

[26] Nasr Eldeen S.K., El-Magd M.A., Khamis A., Ibrahim W.M., Salama A.F. (2018). Anticancer effect of trehalose by modulating oxidative stress, apoptosis and autophagy. AJMS.

[27] Mackeh R., Perdiz D., Lorin S., Codogno P., Poüs C. (2013). Autophagy and microtubules-new story, a old players. J. Cell Sci..

[28] Lombardo D., Kiselev M.A., Magazù S., Calandra P. (2015). Amphiphiles self-assembly: Basic concepts and future perspectives of supramolecular approaches. Adv. Cond. Matter Phys..

[29] Kitamoto D., Morita T., Fukuoka T., Konishi M., Imura T. (2009). Self-assembling properties of glycolipid biosurfactants and their potential applications. Curr. Opin. Colloid Interface Sci..

[30] Zaragoza A., Aranda F.J., Espuny M.J., Teruel J.A., Marqués A., Manresa A., Ortiz A. (2009). Mechanism of membrane permeabilization by a bacterial trehalose lipid biosurfactant produced by Rhodococcus sp. Langmuir.

[31] Zaragoza A., Aranda F.J., Espuny M.J., Teruel J.A., Marques A., Manresa A., Ortiz A. (2010). Hemolytic activity of a bacterial trehalose lipid biosurfactant produced by Rhodococcus sp.: Evidence for a colloid-osmotic mechanism. Langmuir.

[32] Zhang Y., Yang M., Portney N.G., Cui D., Budak G., Ozbay E., Ozkan M., Ozkan S.C. (2008). Zeta potential: A surface electrical characteristic to probe the interaction of nanoparticles with normal and cancer human breast epithelial cells. Biomed. Microdevices.

[33] Gal N., Weihs D. (2012). Intracellular mechanics and activity of breast cancer cells correlate with metastatic potential. Cell Biochem. Biophys..

[34] de Oliveira I.F.A. (2015). Fighting Breast Cancer Using Membrane-Active Peptides. Ph.D. Thesis.

