# Supplementary material for: Bacterial Natural Disaccharide (Trehalose Tetraester): Molecular Modeling and in Vitro Study of Anticancer Activity on Breast Cancer Cells
Source: Polymers (Basel). 2020 Feb 24;12(2):499. doi: 10.3390/polym12020499 (PMC7077702; doi:10.3390/polym12020499)
Supplement: Supplementary file 1 [file polymers-12-00499-s001.pdf]

Supplementary material

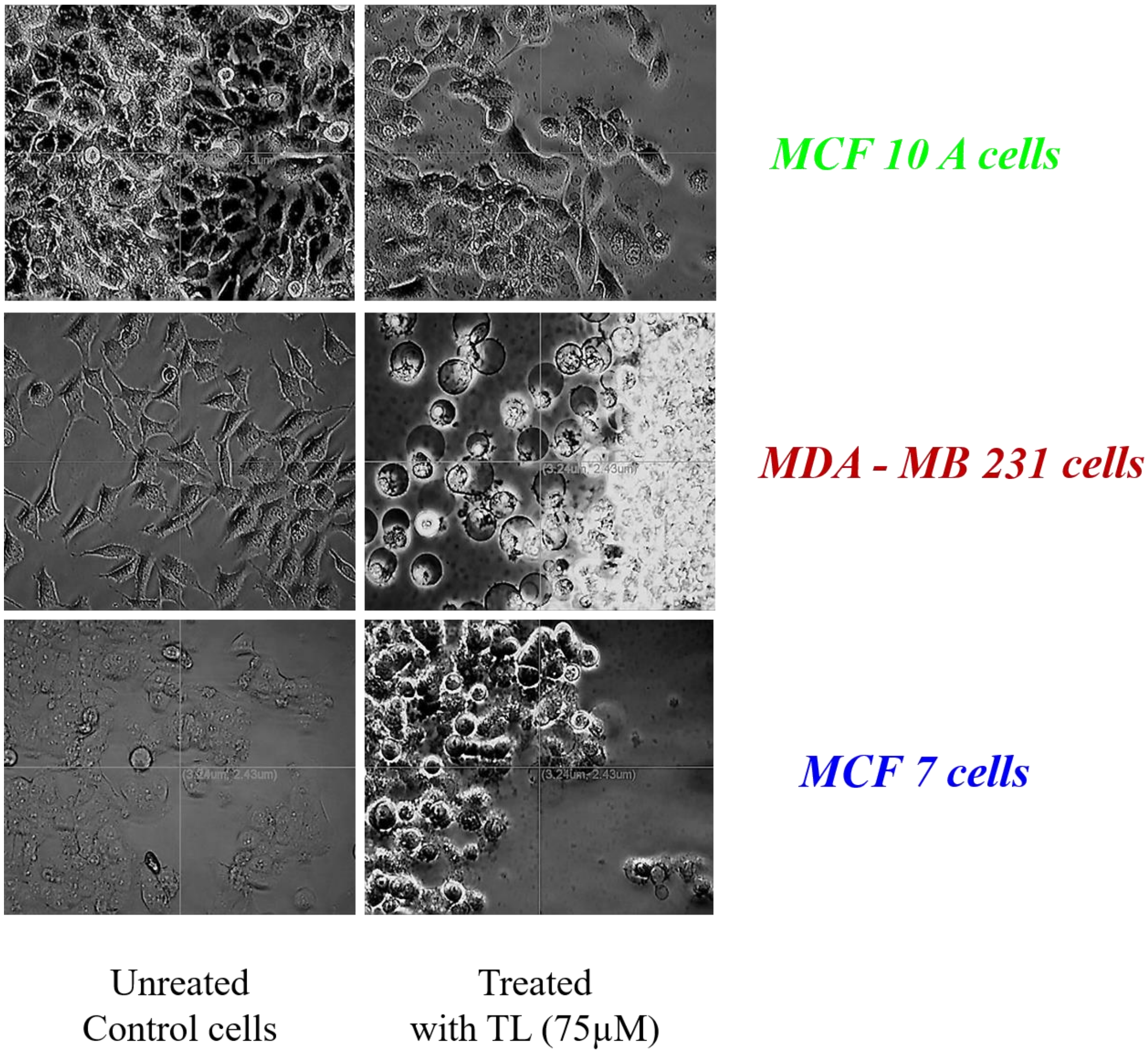

Figure S1. Changes in cell morphology of normal MCF10A cells /top panel/ and cancer cells /MDA-MB231 cells – middle panel; MCF7 cells – bottom panel/ 48 h after treatment with subcytotoxic concentration TL concentration (75 µmol), analyzed by phase-contrast light microscopy. Untreated cells were used as a control.
